# Environmental tobacco smoke exposure is associated with increased levels of metals in children’s saliva

**DOI:** 10.1038/s41370-023-00554-w

**Published:** 2023-05-05

**Authors:** Lisa M. Gatzke-Kopp, Jenna L. Riis, Hedyeh Ahmadi, Hillary L. Piccerillo, Douglas A. Granger, Clancy B. Blair, Elizabeth A. Thomas

**Affiliations:** 1 Department of Human Development and Family Studies, Penn State University, University Park, PA 16802 USA; 2 Department of Psychological Sciences, University of California Irvine, Irvine, CA USA; 3 Institute for Interdisciplinary Salivary Bioscience Research, University of California Irvine, Irvine, CA USA; 4 University Statistical Consulting, LLC, Irvine, CA USA; 5 Johns Hopkins University, Bloomberg School of Public Health, and School of Medicine, Baltimore, MD USA; 6 Department of Population Health, New York University Grossman School of Medicine, New York, NY USA; 7 Department of Epidemiology, University of California Irvine, Irvine, CA USA

**Keywords:** Environmental tobacco smoke, trace metals, biomarker, saliva, cotinine

## Abstract

**Background:**

Exposure to environmental tobacco smoke (ETS) has been associated with detectable levels of cotinine (a nicotine metabolite) in children’s saliva. However, tobacco smoke also contains toxic and essential trace metals, including chromium (Cr), copper (Cu), lead (Pb), manganese (Mn), nickel (Ni) and zinc (Zn).

**Objective:**

The current study examines whether there is a relationship between ETS exposure, as gauged by salivary cotinine, and salivary levels of these metals in a subset (*n* = 238) of children from the Family Life Project.

**Methods:**

Using inductively-coupled-plasma optical emission spectrophotometry, we measured levels of metals in saliva from children at ~90 months of age. Salivary cotinine was measured using a commercial immunoassay.

**Results:**

We found that Cr, Cu, Mn, and Zn were detected in most samples (85–99%) with lower levels of detection for Pb and Ni (9.3% and 13.9% respectively). There were no significant differences in any of the metal concentrations between males and females, nor were levels associated with body mass index, although significant differences in salivary Cr and Mn by race, state and income-to-needs ratio were observed. Children with cotinine levels >1 ng/ml had higher levels of Zn (*b* = 0.401, 95% CI: 0.183 to 0.619; *p* = 0.0003) and Cu (*b* = 0.655, 95% CI: 0.206 to 1.104; *p* = 0.004) compared to children with levels <1 ng/ml, after controlling for multiple confounders, including sex, race, BMI and income-to-needs ratio. Further, we show that children whose cotinine levels were >1 μg/L were more likely to have detectable levels of Pb in their saliva (*b* = 1.40, 95% CI: 0.424 to 2.459; *p* = 0.006) compared to children with cotinine levels <1 ng/ml, also considering confounders.

**Impact statement:**

This is the first study to demonstrate significant associations between salivary cotinine and salivary levels of Cu, Zn and Pb, suggesting that environmental tobacco smoke exposure my be one source of increased children’s exposure to heavy metals. This study also demonstrates that saliva samples can be used to measure heavy metal exposure, and thus serve as a non-invasive tool for assessing a broader range of risk indicators.

## Introduction

Environmental tobacco smoke (ETS) remains a major source of indoor air pollution that can cause major health problems in children, including lower respiratory tract infections, asthma, childhood cancers, behavioral problems and decreased performance in school [1–4]. Although decreases in tobacco smoking, awareness of ETS hazards, and smokefree policies may have contributed to a reduction in ETS exposure in the past few decades, in recent years, the percentage of youth with ETS exposure has remained steady [5]. Tobacco smoke contains thousands of chemical compounds, most of which are harmful to humans [6]. Further, tobacco, cigarette paper, filters, and cigarette smoke are known to contain metals, including chromium (Cr), copper (Cu), lead (Pb), manganese (Mn), nickel (Ni) and zinc (Zn) [7–9]. Accordingly, cigarette smoking may be a substantial source of intake of these metals, not only to the smoker but also to nonsmokers in the form of secondhand smoke (i.e. airborne smoke from burning cigarette and smoker exhalation), and thirdhand smoke (i.e. physical residue that settles onto clothes, furniture and flooring) [10]. Previous work from our laboratory has demonstrated that young children (e.g. 6 to 48 months of age) are capable of absorbing nicotine from environmental exposure at levels commensurate to those seen in active smokers [11].

Many metals that are found in tobacco and/or cigarette smoke are essential trace metals that are typically present in the human body in extremely small quantities (less than 0.01%). These metals are essential for numerous biological and chemical processes, including regulating cellular homeostasis, acting as cofactors for many enzymes and serving as antioxidant molecules [12]. Cu, Mn and Zn are among the most widely studied essential trace metals, while Cr has been questioned as an essential metal, given its known toxic properties [13]. Although these elements play critical biological roles in small amounts, they all can be toxic at sufficiently high levels [14]. Excess amounts of these and other metals in the brain are known to be associated with a variety of neurological conditions, including cognitive impairment, behavioral problems, psychiatric, and motor abnormalities in children [15–22]. Elevated Mn has been associated with deficits in cognitive behavior, motor function, memory and behavioral problems [17, 18, 20, 21]. Levels of Cu and Cr have been found to be significantly associated with verbal IQ scores and visuospatial learning in children and adolescents [15, 19]. Pb is a nonessential, highly neurotoxic heavy metal that is one of the most-widely studied toxic metals. Several studies have demonstrated an impact of Pb exposure on learning and behavioral disorders during early adolescence [19, 23], and have shown direct relations between Pb and psychiatric conditions, such as attention-deficit disorder [24, 25]. Further, environmental co-exposure to Pb, Mn and Cr have also been shown to be important in children, with higher levels of Mn, Pb, and Cr found to be associated with lower IQ scores [22].

Overall the relationships between smoking and metals levels in biological fluids has not been widely investigated, although previous studies have shown that levels of metals in blood and urine are increased in smokers; these studies include findings that adult smokers showed increased levels of Cr in urine [26], Cu levels in serum [27], Zn levels in serum [28] and Pb levels in blood [29] compared to non-smokers. Only two previous studies have reported on measures of metals from saliva of adult smokers vs. non-smokers; [7, 30] however, no studies have specifically examined whether environmental exposure to tobacco smoke (inhalation and ingestion) is associated with increased exposure to metals.

Cotinine, a metabolite of nicotine, is the biochemical marker of choice for quantifying passive exposure to smoke. It is specific to tobacco, with a half-life of about 20 h, and can be assayed by a simple commercially available assay. Salivary levels of cotinine are correlated to those found in blood [31, 32], and provide a non-invasive method of quantifying passive smoke exposure. In this study, we report on the feasibility of measuring essential and toxic metals in saliva samples from a population of young children and demonstrate positive associations between ETS exposure, measured using salivary cotinine, and salivary metal concentrations in this sample.

## Materials and methods

### Participants

This study was reviewed and approved by the Office of Human Research Ethics at the University of North Carolina (IRB #07-0646; #16-2751). Parents provided consent for the collection and analyses of children’s saliva samples. Subjects were recruited as part of the Family Life Project (FLP), a prospective, population-based longitudinal study that recruited approximately 1300 families at the time of their child’s birth, between September 1st 2003 and August 31st 2004. Families were recruited from three non-urban counties in Pennsylvania and three counties in North Carolina and were oversampled for poverty. A detailed characterization of the sampling plan and study has been published elsewhere [33]. This study uses data and specimens collected from home visits when children were ~90 months of age, with parents or primary caregivers providing consent. For the current study, archived saliva samples with sufficient volume (see below) were used, resulting in a subsample of the original cohort consisting of *n* = 238 child participants. Children in this subsample did not differ from the non-included participants with respect to state of residence (36.5% vs. 41.4% from Pennsylvania (c^2^ (1, *N* = 238)=1.89, *p* = .16), child race (44.9% vs. 39.9% African American (χ^2^ (1, N = 238) = 2.06; *p* = 0.15) or child sex (50.8% vs. 49.1% male χ^2^ (1, *N* = 238) = 0009, *p* = 0.97), but were less likely to have been identified as low-income at the time of enrollment (34.4% vs. 76.6% poor (χ^2^ (1, *N* = 238) = 161.1, *p* = <0.0001).

### Saliva sample collection

An infrared thermometer was used to verify that the child did not have a fever prior to collecting saliva. Saliva was collected approximately 1 h into the home visit protocol, ensuring that the child had not consumed any food or drink in that interval. Unstimulated whole saliva samples were collected from children using the passive drool method [34] during the 90 month visit to participants’ homes. At the time of collection, samples were frozen at −20 °C and then transferred to the Institute for Interdisciplinary Salivary Bioscience Research at the University of California, Irvine for archiving at −80 °C until used. At the time of use, saliva samples were thawed and centrifuged (5000 g; 10 min; 4 °C) to remove insoluble material and cellular debris. Supernatants were collected and used for other assays separate from the current study. For the current study, samples with at least 0.8 ml of saliva remaining were used, to allow for enough volume to run the metals analysis.

### Salivary metals analysis

Levels of metals in saliva were measured using Inductively Coupled Plasma Optical Emission Spectrometry (ICP-OES) (Avio200; Perkin Elmer, Waltham, MA, USA) using the radial viewing window [35]. All glassware was treated with 10% (volume/volume; v/v) HNO_3_ before use and then rinsed with deionized water. Prior to analysis, the ICP-OES was aligned with 0.1% manganese. Samples were diluted 1:7 in 2% HNO3. The ICP-OES was operated with argon carrier flow rate of 0.5 L/min, plasma gas flow rate of 15 L/min, sample flow and elusion rate of 1.51 L/min, and peristaltic pump speed of 100 rpm. Optimal wavelengths for each element were chosen by using software that enabled selection of peaks that lack interference. The resulting suitable wavelengths were selected for each element: Cr (267.716), Cu (327.393), Mn (257.610), Ni (231.604), Pb (220.353), Zn (206.200). Metals output was normalized to the internal standard, 0.1% Yttrium, and compared to standard curves generated for each metal, which were prepared from stock solutions of 1000 ppm. Yttrium recovery values for all samples fell between 89% and 106%. Standard curves for each element showed linearities r^2^ > 0.99. Known control samples were run once every 20 samples. The analytical detection limits for all biomarkers were based on repeated measurements of procedural blanks on four separate analysis days and these are provided in Table 1.

**Table 1:** Levels of metals and cotinine in saliva of child participants.

	# detected	LLoD (μg/L)	# < LLoD *n* (%)	Mean ± S.D. (μg/L)	Median (μg/L)	Range (μg/L)
Chromium (Cr)	236	0.140	2 (0.8%)	7.39 ± 2.7	7.12	2.40–22.46
Copper (Cu)	201	0.45	37 (15.5%)	34.37 ± 71.7	15.66	2.31–844.17
Lead (Pb)	22	2.20	216 (90.7%)	18.12 ± 6.5	17.66	4.50–33.68
Manganese (Mn)	213	0.073	25 (10.5%)	9.81 ± 15.2	4.48	0.59–122.53
Nickel (Ni)	33	1.32	205 (86.1%)	26.32 ± 31.6	16.20	9.39–186.23
Zinc (Zn)	237	0.18	1 (0.4%)	61.11 ± 60.6	45.02	8.58–565.98
Cotinine	185	0.15	53 (22.2%)	7.83 ± 64.2	0.93	0.16–851.70

The numbers shown reflect the number of samples with concentrations of the analyte above the lower limit of detection (LLoD) of the assay. The total sample number = 238. The ranges shown are those values that were detected above the LLoD. *SD* standard deviation. The mean and median levels of metals shown do not include those values below LLoD.

Prior to the analysis of study samples, we first tested whether the levels of a spiked-in, known amount of each metal (500 ug/L) varied according to saliva collection method (passive drool vs. swab), saliva processing method (centrifuged (as above) vs. uncentrifuged), storage conditions (−20 °C, 4 °C, room temperature) over 24 h, or the number of freeze-thaw cycles (1–3). This was carried out on a separate group of control adult subjects (*n* = 5). Comparisons of metals levels were made between the given conditions using Student’s *t*-tests (unpaired; two-tailed) (GraphPad Prism).

### Salivary cotinine

Salivary cotinine has been shown to correlate well with levels found in urine and blood [36, 37], promoting the use of saliva as a reliable fluid to estimate ETS. Salivary cotinine was assayed in duplicate using a commercially-available, ELISA kit (Salimetrics LLC, Carlsbad, CA) following the manufacturer’s protocol. The assay has a test volume of 20 µL, range of standards from 0.8 to 200 μg/L, and lower limit of detection of 0.15 μg/L. The intra-assay precision was determined from the mean of 10 replicates and resulted in acoefficients of variation (CV) = 7.10%). Inter-assay precision was determined from eight separate runs returned a CV of 9.1%). Cotinine high and low controls were run on every assay plate. Per the manufacturer’s protocol, the recovery of spiked in cotinine in the saliva matrix was 94.1 to 114% (Salimetrics LLC, Carlsbad, CA)

### Salivary flow rate

Salivary flow rate (ml/min) was calculated for each subject using researcher-reported saliva collection durations and estimated sample volumes determined by weight.

### Covariates

Child age, sex, body mass index (BMI) and race were reported by primary caregivers. Race was dichotomized as African American vs. not African American (Table 2). Family income-to-needs ratio was assessed at the 90 month visit using parent/caregiver report of total household income and the number of adults and children in the household. State of the participants’ residence was also recorded.

**Table 2:** Sociodemographic characteristics of study participants (*N* = 238).

Characteristic	Mean ± SD or *n* (%)
Age (months)	86.98 +/− 3.32
Sex	
Male	121 (50.8%)
Female	117 (49.2%)
Race (African American)	107 (44.9%)
BMI	18.00 +/− 4.39
Family INR	1.83 +/− 1.62
State of residence (NC)	151 (63.4%)

*BMI* Body mass index. *INR* income-to-needs ratio. Subjects not from North Carolina (NC) were from Pennsylvania.

### Statistical analysis

All statistical tests were carried out using GraphPad Prism, version 9.5. Prior to conducting analyses, measures of analytes that were below the limit of the instrument detection were replaced with detection thresholds for that analyte, except for Pb and Ni, both of which had >80% of observations below the detection threshold. (See Table 1 for detection thresholds and the numbers of determinations detected). Kolmogorov-Smirnov and Shapiro-Wilk normality tests revealed that all analytes were not normally distributed. For cotinine, samples were then categorized as those subjects with low ETS exposure (<1 ng/ml), coded as “0”, or those with high ETS exposure (>1 ng/ml), coded as “1” (http://www.cdc.gov/exposurereport/). Differences in metal concentrations by sex, race, and state were examined using the Mann–Whitney *U* test. Associations between salivary metal concentrations and flow rate, BMI, and income-to-needs ratios were assessed using Spearman correlations. Intercorrelations among salivary metal concentrations were examined using Spearman correlations.

The associations between cotinine and salivary levels of Cu, Cr, Mn and Zn were analyzed using multiple linear regression with each salivary metal as the dependent variable and cotinine as the independent variable, while controlling for the above-mentioned covariates, sex, race, BMI and income-to-needs ratio. To improve normality, salivary metals levels were ln-transformed before regression analyses. Due to the low detection rates for Ni and Pb, these two metals were dichotomized based on the detection rate (“0” for not detected and “1” for a detected value) and analyzed using logistic regression. Inspection of standardized residuals, QQ plots and homoscedasticity plots was used to ensure no violation of the test assumptions. For all analyses, an alpha level of .05 was used to establish statistical significance and Bonferroni corrected alpha levels (0.05/the number of tests conducted) were applied to account for multiple testing.

## Results

### Participant demographics

The characteristics of study participants are summarized in Table 2. The average age ± SD of children in this study was 86.98 +/− 3.32 months, or approximately 7 years of age. Approximately half of the sample was male and half were parent-identified as African American. More than half of the participants (63.4%) were from North Carolina, with the remainder from Pennsylvania (Table 2). 34.4% of the subjects were from families below the poverty line, defined by an income-to-needs ratio of ≤1.

### Salivary metals characteristics

#### Assay specifics

We measured levels of metals previously associated with tobacco smoke, including Cr, Cu, Mn, Ni, Zn and Pb, in saliva specimens from this subsample of children using ICP-OES. To establish the validity and reliability of the salivary metal measurements, we first tested whether the levels of a known amount of each metal varied according to saliva collection method (passive drool vs. swab), saliva processing method (centrifuged vs. uncentrifuged), storage conditions (−20 °C, 4 °C, room temperature), or the number of freeze-thaw cycles using separate saliva samples collected from adult controls. We found no evidence that the levels of the salivary metals varied significantly by any of these factors, with the exception of Zinc which showed higher recovery in saliva samples obtained via swab collection (Supplimentary Fig. 1).

#### Metals levels in FLP children

The mean and median salivary levels of each metal are shown in Table 1. Essential trace metals, Cu, Mn and Zn, as well as Cr, were detected in almost all participants (89.5–99.2%) (Table 1). Toxic metals, Pb and Ni were detected in only 9.3 and 13.9% of our samples, respectively, with the remaining samples being below the detection limit of the instrument. Many of the metals were highly positively intercorrelated, with the divalent cations (Cu, Mn and Zn) showing the strongest correlations with one another (Supplimentary Table 1). There was no effect of salivary flow rate on any of the metals or cotinine levels (Spearman correlation: rho value range 0.19–0.59; *p*-value range = 0.245 to 0.715) (data not shown).

#### Salivary metals and children/family factors

Comparisons of metal concentrations across demographic characteristics are reported in Table 3. There were no differences in concentrations of any of the metals between males and females, nor were levels of any metal associated with BMI. Cr levels were higher among African-American children compared to non-African-American children and among those living in North Carolina compared to Pennsylvania. It is important to note, however, that there is a statistically significant association between race and state in this sample, with the majority of African American participants residing in North Carolina (Chi square test, *p* < 0.0001). The median levels of Mn and Pb were lower among African-American children compared to non African-American children, and these differences were not associated with state of residence. Negative associations were observed between family income-to-needs ratio and both Cr and Zn levels (Table 3).

**Table 3:** Associations between salivary metal concentrations and child and family characteristics.

	Child Sex	State	Child race	Child BMI	Family INR:
	MD	MD	MD		
	*p*-value	*p*-value	*p*-value	rho, *p*-value	rho, *p*-value
Cr	MD_F_ = 7.18	MD_NC_ = 7.68	MD_NB_ = 6.04		
	MD_M_ = 6.99	MD_PA_ = 5.76	MD_BL_ = 7.86		
	0.396	**0.017**	<**0.0001**	0.117, 0.074	**−0.16, 0.015**
Cu	MD_F_ = 11.4	MD_NC_ = 12.6	MD_NB_ = 13.4		
	MD_M_ = 12.7	MD_PA_ = 9.40	MD_BL_ = 11.5		
	0.373	0.482	0.111	−0.011, 0.868	−0.089, 0.168
Mn	MD_F_ = 3.44	MD_NC_ = 4.28	MD_NB_ = 5.34		
	MD_M_ = 3.29	MD_PA_ = 3.26	MD_BL_ = 3.07		
	0.774	0.189	**0.007**	−0.12, 0.065	0.081, 0.213
Zn	MD_F_ = 47.4	MD_NC_ = 46.7	MD_NB_ = 40.8		
	MD_M_ = 40.9	MD_PA_ = 38.7	MD_BL_ = 46.7		
	0.512	0.165	0.171	0.033, 0.607	**−0.299, 0.0004**
Pb	MD_F_ = 18.6	MD_NC_ = 15.3	MD_NB_ = 20.3		
	MD_M_ = 17.7	MD_PA_ = 8.7	MD_BL_ = 14.7		
	0.791	0.869	**0.038**	0.181, 0.387	0.162, 0.438
Ni	MD_F_ = 15.2	MD_NC_ = 15.4	MD_NB_ = 18.8		
	MD_M_ = 17.8	MD_PA_ = 21.2	MD_BL_ = 15.0		
	0.539	0.366	0.645	−0.192, 0.262	0.039, 0.821

*BMI* body mass index; In/Ratio, family income-to-needs ratio. State refers to the child state of residence. Correlations to BMI and income-to-needs ration (INR) were determined by Spearman correlation. Differences in median (MD) levels by sex, state and race were determined using the Mann–Whitney test. *BL* Black/African American; *NB* not Black/African American. For these associations, values that were below the LLoD were substituted with the LLoD values (Table 2) prior to the calculations, except for Pb and Ni, which were present at undetectable levels in >80% of children’s samples. The unit for each metal was in μg/L.

Statistically significant values are in bold.

### Associations between salivary cotinine and metal levels

The median salivary cotinine level in this subsample was 0.93 ng/ml (range 0.16–851.7 ng/ml), with a mean level of 7.83 μg/L +/− 64.2 (S.D) (Table 1). As presented in Table 4, children with salivary levels of cotinine >1 ng/ml had higher salivary levels of Zn (*b* = 0.401, 95% CI: 0.183 to 0.619; *p* = 0.0003) and Cu (*b* = 0.655, 95% CI:0.206 to 1.104; *p* = 0.0044), compared to children with cotinine levels <1 ng/ml, after controlling for multiple confounders, including sex, race, BMI and income-to-needs ratio. Cotinine levels were not strongly associated with salivary levels of Cr (*b* = 0.093, 95% CI: −0.0015 to 0.187; *p* = 0.054) and Mn (*b* = 0.367, 95% CI: −0.093 to 0.826; *p* = 0.117), with 95% CI that included 0 (Table 4). For salivary Pb and Ni, values were categorized according to detection rate, and accordingly, we used multiple logistic regression to test for associations with cotinine. From these analyses, we found that children with cotinine levels >1 ng/ml were more likely to have detectable levels of Pb (*b* = 1.40, 95% CI: 0.424 to 2.459; *p* = 0.006) compared to children with cotinine levels <1 ng/ml, also considering confounders, sex, race, BMI and income-to-needs ratio (Table 5). The relationship between salivary cotinine and Ni was less striking (*b* = 0.288, 95% CI: −0.543 to 1.103; *p* = 0.488) (Table 5).

**Table 4:** Associations between salivary cotinine and salivary metals.

	Ln Zn	Ln Cu	Ln Cr	Ln Mn
	*b*, 95% CI	*b*, 95% CI	*b*, 95% CI	*b*, 95% CI
Cotinine*	**0.401, (0.183 to 0.619)**	**0.655, (0.206 to 1.104)**	0.093, (−0.0015 to 0.187)	0.367, (−0.0933 to 0.826)
Sex	0.022, (−0.182 to 0.225)	(−)0.139, (−0.559 to 0.281)	0.032, (−0.056 to 0.121)	0.218, (−0.537 to 0.322)
BMI	(−)0.0035, (−0.032 to 0.025)	(−)0.02, (−0.079 to 0.038)	0.004, (−0.008 to 0.016)	0.030, (−0.107 to 0.014)
Race	0.28, (−0.194 to 0.251)	0.433, (−0.025 to 0.892)	**0.236, (0.139 to 0.333)**	0.238, (−0.101 to 0.838)
INR	(−)0.039, (−0.139 to 9.6e-005)	0.0072, (−0.213 to 0.073)	0.011, (−0.018 to 0.041)	0.074, (−0.073 to 0.221)

Associations were determined by multiple linear regression. Statistically significant findings are shown in bold font. Metals outcome values were ln-transformed prior to analysis. *cotinine was coded 0 for values less than 1 ng/ml and 1 for >1 ng/ml. *BMI* body mass index.

*INR* income-to-needs ratio.

**Table 5:** Associations between salivary cotinine and detection rates for salivary Pb and Ni.

	Pb	Ni
	*b*, 95% CI	*b*, 95% CI
Cotinine*	**1.40, (0.424 to 2.459)**	**0.288, (-0.5431 to 1.103)**
Sex	(−)0.302, (−1.254 to 0.623)	(−)0.309, (−1.091 to 0.4506)
BMI	(−)0.107, (−0.285 to 0.037)	(−)0.035, (−0.1618 to 0.07423)
Race	(−)0.761, (−1.764 to 0.201)	**(−)0.886, (−1.738 to −0.07189)**
INR	(−)0.044, (−0.421 to 0.237)	(−)0.027, (−0.3000 to 0.2014)

Associations were determined by multiple logistic regression. Statistically significant findings are shown in bold font. Metals outcome values were dichotomized by detection limit. *cotinine was coded 0 for values less than 1 ng/ml and 1 for >1 ng/ml. *BMI* body mass index. *INR* income-to-needs ratio.

## Discussion

In this study, we demonstrated that essential trace elements (Cu, Cr, Mn and Zn) were readily detected in saliva samples from healthy young children from the FLP cohort, while toxic metals (Pb and Ni) were present in fewer than 15% of children. Notably, Cr, which can be considered both an essential and toxic metal, was detected in nearly all samples. Although studies have documented the presence of each of these metals in tobacco products including cigarettes, less research has examined whether they are transmitted through second- and third-hand exposure. Our findings demonstrate that levels of cotinine, a measure of ETS, were positively associated with salivary levels of Zn, Cu and Pb, supporting the supposition that ETS exposes children to the full range of associated toxins in addition to nicotine. Notably, these studies are the first to exclusively use saliva samples to demonstrate these effects.

While most previous studies measuring levels of toxic and essential trace elements have been carried out in blood, an increasing number of studies have demonstrated that saliva may be a reliable indicator of environmental and occupational exposures to trace metals [38–41]. Although some studies have revealed significant correlations between serum and saliva for certain metals, such as Mn and Cu [41, 42], levels of other metals, such as Zn, Cd or Pb, in saliva were not significantly correlated with blood counterparts [39, 41, 42], however one study demonstrated significant associations between salivary and whole blood Pb in an exposed population [43]. Thus, variations in saliva metal levels may mirror, only partly, the changes of the concentrations of these metals in blood. Due to their electric charge, metal ions cannot enter cells by simple diffusion, and instead necessitate a dedicated transport system to cross cell membranes [44], hence, this is thought to be one reason why some metals are not correlated between saliva and blood [45]. Therefore, saliva is not a simple surrogate for blood or other body fluids, but rather it has its own distribution of trace metals. While there are guidelines for acceptable levels of most metals measured in blood (https://www.atsdr.cdc.gov/), there are currently no official guidelines for salivary metal levels in humans [46]. Nonetheless, measurements of trace metals in saliva are thought to provide a non-invasive alternative to blood sampling, that can reflect environmental exposures, as previously suggested [7].

Levels of metals measured in this study were comparable to previous studies measuring salivary metals in healthy adult and child populations. A previous study carried out in children reported a median salivary Mn level of 4.91 μg/L [38], which is highly consistent with our median Mn level of 4.48 μg/L. The mean salivary Mn concentration detected in our study was 9.81 μg/L, which is similar to previous reports in adults [45, 47] and in children [41, 48]. These findings suggest that our sample is likely comparable with regard to dietary and environmental sources of Mn. In contrast, the mean (61.1 μg/L) and median (45.02 μg/L) Zn levels observed were slightly lower than levels previously reported in a sample of 29 healthy non-smoking adults (mean 75.3 μg/L, median 57.3 μg/L) [47]. However, the range of Zn observed in that study was 0.3 to 124.3, whereas the range observed in our study was much larger (8.58–565.98) possibly suggesting that a higher magnitude of exposure observed for some children might be related to their ETS exposure. Cu also showed a wide range in observed levels (2.31–844.17) in our sample. Past studies measuring salivary Cu have been inconsistent, with some only reporting the means and others reporting only the medians, making studies difficult to compare. One study reported a median Cu level of 21.6 μg/L [38], which is only slightly higher than the levels measured in the current study (15.7 μg/L). However, our mean level for Cu (34.4 μg/L) is markedly higher than that reported in other studies on adults (8.2 μg/L [47]) and children (2.05 μg/L [48]). Previous studies of Cr have reported a mean range of Cr = 0.327 to 1.13 μg/L) [45, 49, 50], much lower than the values identified in our study (mean range 2.40–22.46 μg/L).

Among these essential metals, levels of salivary cotinine were significantly associated with salivary levels of Zn and Cul The exponentiated parameter coefficients for Zn and Cu were 1.49 and 1.93, respectively, indicating an 49% and 93% increase in metals levels with respect to a one unit increase in exposure to cotinine. Table 4 shows that covariables, including sex, race, BMI and income-to-needs ratio, did not appear to contribute to these associations. Cotinine did not appear to be related to substantial changes in the levels of salivary Cr and Mn, (95% CI: −0.0015 to 0.187 and 95% CI: −0.093 to 0.826, for Cr and Mn, respectively), and with only borderline statistical significance. However, it is important to remember that statistical significance does not imply biologically nor clinically significant associations, and that more research is needed to know what levels of exposure relate to meaningful changes in health outcomes. Future research could examine whether changes in cotinine across time are associated with correlated changes in metals concentration, for instance following successful smoking cessation. It is important to note, however, that cotinine and heavy metals differ with regard to the half-life in the body. Cotinine is metabolized relatively quickly, and thus salivary values reflect very recent, acute exposure. In contrast, heavy metals may be excreted much more slowly. Blood levels may reflect exposure over several months, and many heavy metals are known to accumulate in various organ tissues [51] As such, examining temporal covariation between cotinine and heavy metals requires careful consideration of the sampling frequency.

In contrast to the essential metals, Pb was only detected in <10% of the samples. The prevalence of Pb exposure, as well as the range of concentrations (4.50–33.68 ug/L) was similar to a study of salivary Pb levels from another study on healthy children from an industrialized region in Germany (range <1.5–47.0 ug/L) [52]. Several studies have suggested that ETS might be associated with increased levels of Pb in the blood of children [53–55]. In one study, participants in the highest quartile of serum cotinine (≥0.44 μg/L) had 28% higher blood lead levels than had those in the lowest quartile (<0.03 μg/L). Similarly, blood lead levels were 14% and 24% higher in children who lived with 1 or with 2 or more smokers, respectively, than they were in children living with no smokers [53]. In another study, the geometric mean blood lead levels were 38% higher in children with high cotinine levels (>0.563 μg/L) compared with children who had low cotinine levels (<0.104 μg/L) [55]. In accordance with these reports, we found that children with cotinine levels, indicative of ETS exposure (>1 ng/ml) had higher odds of detecting Pb in saliva compared to children with cotinine levels below 1 ng/ml (i.e. non-ETS exposed), with exponentiated parameter coefficient of 4.05, indicating a four-fold increase in Pb levels per one-unit increase in cotinine. These findings might suggest that Pb exposure accompanies more substantial levels of ETS exposure.

Besides ETS, other potential environmental exposures to these metals could include environmental contamination, air pollution due to traffic or industrial emissions, metal contaminants from homes, diet and poor oral health. It is possible that the associations between Cr, Mn, and Pb with child race reflects health disparities in environmental exposures. In particular, Cr, Ni and Mn exposures are known to be widespread in contaminated air, soil and water [38, 40, 56]. For example, studies have shown that Mn concentration in water and soil were significantly associated with Mn in saliva [38, 40], however similar findings were not found with Cr and Cu [38]. Another study specifically demonstrated higher salivary concentrations of Mn in adult welders compared to controls, an association attributed to airborne Mn levels among study populations [41]. In addition, oral health issue, including dental cavities, could possibly affect salivary metals levels in these children. Previous reports have demonstrated some links between salivary metals and dental caries in children [48, 57–59], although findings were not consistent. One study reported positive correlations between dental caries and salivary Cu and Zn [59], although another study observed a negative relationship between dental caries and salivary Cu [58]. Several other studies showed no associations with dental caries and salivary Zn, Fe or Mn [48, 57, 58].

Overall, these findings support the reliable measurements of toxic and essential metals in salivary samples from children, which could provide the basis for further comparisons between salivary metals and behavioral, cognitive or other clinical measures. Our results also suggest that second- and third-hand exposure to cigarette smoking could contribute as one source of exposure to Cr, Cu, Zn, and Pb. The recent rise in electronic cigarette use (e.g. vaping) has been cast as a healthier alternative to tobacco-based products. Studies of common products used for vaping report varying levels of toxic metals in the liquids, with some indication that the metals present in the aerosolized smoke are a function of the device itself (e.g. the metal composition of the vaping device or heating coils) [60, 61]. Importantly, in these instances the metals were detected in the aerosolized vapors, indicating that any substance contained within the product is just as capable of being transmitted second- (through airborne routes) and third-hand (surface residue contamination) including the nicotine itself [60]. As such, public health messaging should continue to advance the information that no form of smoking is safe in areas where young children could be exposed.

Although salivary levels of cotinine are correlated to those found in blood [31, 32, 37] and urine [36], measures of bodily fluids are subject to shorter half-life window for detection relative to hair or nails that would capture the magnitude of sustained exposure [62]. Levels of cotinine in urine are more sensitive, being 4–6 times higher than salivary cotinine levels [63], nonetheless, salivary saliva cotinine is considered to be an excellent exposure marker of cigarette smoke without invasive methods for collecting body fluids. We have previously shown a high level of stability in the severity of ETS exposure in a different, but overlapping, subsample of FLP children across four timepoints between 6 and 48 months of age [11]. As such, saliva could be an effective, and non-invasive way to screen children for exposure. Research has demonstrated that policies enacted to reduce smoking in publicly occupied spaces results in significantly lower levels of cotinine detected in children [5].

The findings reported here must be considered in light of the broader sample characteristics. The FLP sample was originally oversampled for poverty (76.6% of the full sample). However, the subsample with sufficient remaining saliva volumes was less reflective of poverty, with only 34.4% of the current subsample characterized by low income. While we found statistically significant negative correlations between salivary Cr and Zn levels with income-to-needs ratio in this subsample, this relationship might be different if applied to the entire FLP cohort of nearly 1300 children. Additionally, we have previously shown that the prevalence and magnitude of ETS exposure in this sample is higher than the national average [11], which may have facilitated the detection of associations that may only emerge at higher levels of exposure. Ultimately it will be important to consider the range of environmental sources that could contribute to this exposure, and more research is needed to determine the clinical implications of salivary levels of these metals to better guide screening and prevention practices.

This is the first study to demonstrate significant associations between salivary cotinine and salivary levels of Cu, Zn and Pb, providing support for ETS as a modifiable source of metals exposure in children. Further, we suggest that saliva sampling represents a major advance in methodology for monitoring ETS exposures in young children. Saliva has been increasingly utilized in adults to assess a broad array of environmental exposures and biomarkers, providing a relatively simple tool for comprehensive assessments of the human exposome [64–66]. Our current studies specifically extend these arguments to ETS and exposures to children.

### Supplementary Information

Supplementary Information

## Data Availability

The datasets generated and analyzed during the current study are available from the corresponding author on reasonable request.

## References

[1] Best D (2009). From the American academy of pediatrics: technical report—secondhand and prenatal tobacco smoke exposure. Pediatrics.

[2] Carlsen KH, Carlsen KC (2008). Respiratory effects of tobacco smoking on infants and young children. Paediatr Respir Rev.

[3] Hwang SH, Hwang JH, Moon JS, Lee DH (2012). Environmental tobacco smoke and children’s health. Korean J Pediatr.

[4] Gatzke-Kopp L, Willoughby MT, Warkentien S, Petrie D, Mills-Koonce R, Blair C (2020). Association between environmental tobacco smoke exposure across the first four years of life and manifestation of externalizing behavior problems in school-aged children. J Child Psychol Psychiatry.

[5] Brody DJ, Lu Z, Tsai J. Secondhand smoke exposure among nonsmoking youth: United States, 2013–2016. NCHS Data Brief 2019:1–8. 31442196

[6] Talhout R, Schulz T, Florek E, van Benthem J, Wester P, Opperhuizen A (2011). Hazardous compounds in tobacco smoke. Int J Environ Res Public Health.

[7] Kim YJ, Kim YK, Kho HS (2010). Effects of smoking on trace metal levels in saliva. Oral Dis.

[8] Chiba M, Masironi R (1992). Toxic and trace elements in tobacco and tobacco smoke. Bull World Health Organ.

[9] Kim SW, Jung SW, Lee JG, Joo JH, Lee JH, Lee KJ (2019). The exposure level of environmental harmful substances related to the secondhand smoke in Korean non-smoker adults: data from the second Korean National Environmental Health Survey (KoNEHS 2012-2014): a cross-sectional study. Ann Occup Environ Med.

[10] Bush D, Goniewicz ML (2015). A pilot study on nicotine residues in houses of electronic cigarette users, tobacco smokers, and non-users of nicotine-containing products. Int J Drug Policy.

[11] Gatzke-Kopp LM, Willoughby MT, Warkentien SM, O’Connor T, Granger DA, Blair C (2019). Magnitude and chronicity of environmental smoke exposure across infancy and early childhood in a sample of low-income children. Nicotine Tob Res.

[12] Aschner JL, Aschner M (2005). Nutritional aspects of manganese homeostasis. Mol Asp Med.

[13] Vincent JB (2013). Chromium: is it essential, pharmacologically relevant, or toxic?. Met Ions Life Sci.

[14] O W (2004). What are trace elements? Their deficiency and excess states. JMAJ.

[15] Bauer JA, Devick KL, Bobb JF, Coull BA, Bellinger D, Benedetti C (2020). Associations of a metal mixture measured in multiple biomarkers with IQ: evidence from Italian adolescents living near ferroalloy industry. Environ Health Perspect.

[16] Chasapis CT, Ntoupa PA, Spiliopoulou CA, Stefanidou ME (2020). Recent aspects of the effects of zinc on human health. Arch Toxicol.

[17] Guilarte TR (2013). Manganese neurotoxicity: new perspectives from behavioral, neuroimaging, and neuropathological studies in humans and non-human primates. Front Aging Neurosci.

[18] Menezes-Filho JA, Carvalho CF, Rodrigues JLG, Araújo CFS, Dos Santos NR, Lima CS (2018). Environmental co-exposure to lead and manganese and intellectual deficit in school-aged children. Int J Environ Res Public Health.

[19] Rechtman E, Curtin P, Papazaharias DM, Renzetti S, Cagna G, Peli M (2020). Sex-specific associations between co-exposure to multiple metals and visuospatial learning in early adolescence. Transl Psychiatry.

[20] Zoni S, Lucchini RG (2013). Manganese exposure: cognitive, motor and behavioral effects on children: a review of recent findings. Curr Opin Pediatr.

[21] Rodrigues JLG, Araújo CFS, Dos Santos NR, Bandeira MJ, Anjos ALS, Carvalho CF (2018). Airborne manganese exposure and neurobehavior in school-aged children living near a ferro-manganese alloy plant. Environ Res.

[22] Carvalho CF, Oulhote Y, Martorelli M, Carvalho CO, Menezes-Filho JA, Argollo N (2018). Environmental manganese exposure and associations with memory, executive functions, and hyperactivity in Brazilian children. Neurotoxicology.

[23] Al Osman M, Yang F, Massey IY (2019). Exposure routes and health effects of heavy metals on children. Biometals.

[24] Froehlich TE, Anixt JS, Loe IM, Chirdkiatgumchai V, Kuan L, Gilman RC (2011). Update on environmental risk factors for attention-deficit/hyperactivity disorder. Curr Psychiatry Rep.

[25] Daneshparvar M, Mostafavi SA, Zare Jeddi M, Yunesian M, Mesdaghinia A, Mahvi AH (2016). The role of lead exposure on attention-deficit/ hyperactivity disorder in children: a systematic review. Iran J Psychiatry.

[26] Chen CJ, Shih TS, Chang HY, Yu HS, Wu JD, Sheu SC (2008). The total body burden of chromium associated with skin disease and smoking among cement workers. Sci Total Environ.

[27] Kromhout D, Wibowo AA, Herber RF, Dalderup LM, Heerdink H, de Lezenne Coulander C (1985). Trace metals and coronary heart disease risk indicators in 152 elderly men (the Zutphen study). Am J Epidemiol.

[28] Khabour OF, Alzoubi KH, Al-Sheyab NA, Azab MA, Massadeh AM, Alomary AA (2018). Plasma and saliva levels of three metals in waterpipe smokers: a case control study. Inhal Toxicol.

[29] Mannino DM, Homa DM, Matte T, Hernandez-Avila M (2005). Active and passive smoking and blood lead levels in U.S. adults: data from the third national health and nutrition examination survey. Nicotine Tob Res.

[30] Nizamani P, Afridi HI, Kazi TG, Talpur FN, Baig JA (2019). Essential trace elemental levels (zinc, iron and copper) in the biological samples of smoker referent and pulmonary tuberculosis patients. Toxicol Rep.

[31] Jarvis MJ, Primatesta P, Erens B, Feyerabend C, Bryant A (2003). Measuring nicotine intake in population surveys: comparability of saliva cotinine and plasma cotinine estimates. Nicotine Tob Res.

[32] Parzynski CS, Jaszyna-Gasior M, Franken FH, Moolchan ET (2008). Measuring nicotine intake among highly-dependent adolescent smokers: comparability of saliva and plasma cotinine concentrations. Pharm Biochem Behav.

[33] Vernon-Feagans L, Cox M (2013). The family life project: an epidemiological and developmental study of young children living in poor rural communities. Monogr Soc Res Child Dev.

[34] Granger DA, Fortunato CK, Beltzer EK, Virag M, Bright MA, Out D (2012). Focus on methodology: salivary bioscience and research on adolescence: an integrated perspective. J Adolesc.

[35] Nowak S, Künnemeyer J, Terborg L, Trümpler S, Günsel A, Wiesmüller GA (2015). Analysis of whole blood samples with low gas flow inductively coupled plasma-optical emission spectrometry. Anal Bioanal Chem.

[36] Stragierowicz J, Mikołajewska K, Zawadzka-Stolarz M, Polańska K, Ligocka D (2013). Estimation of cutoff values of cotinine in urine and saliva for pregnant women in Poland. Biomed Res Int.

[37] Avila-Tang E, Al-Delaimy WK, Ashley DL, Benowitz N, Bernert JT, Kim S (2013). Assessing secondhand smoke using biological markers. Tob Control.

[38] Butler L, Gennings C, Peli M, Borgese L, Placidi D, Zimmerman N (2019). Assessing the contributions of metals in environmental media to exposure biomarkers in a region of ferroalloy industry. J Expo Sci Environ Epidemiol.

[39] Barbosa F, Corrêa Rodrigues MH, Buzalaf MR, Krug FJ, Gerlach RF, Tanus-Santos JE (2006). Evaluation of the use of salivary lead levels as a surrogate of blood lead or plasma lead levels in lead exposed subjects. Arch Toxicol.

[40] Ntihabose R, Surette C, Foucher D, Clarisse O, Bouchard MF (2018). Assessment of saliva, hair and toenails as biomarkers of low level exposure to manganese from drinking water in children. Neurotoxicology.

[41] Wang D, Du X, Zheng W (2008). Alteration of saliva and serum concentrations of manganese, copper, zinc, cadmium and lead among career welders. Toxicol Lett.

[42] Bales CW, Freeland-Graves JH, Askey S, Behmardi F, Pobocik RS, Fickel JJ (1990). Zinc, magnesium, copper, and protein concentrations in human saliva: age- and sex-related differences. Am J Clin Nutr.

[43] Staff JF, Harding AH, Morton J, Jones K, Guice EA, McCormick T (2014). Investigation of saliva as an alternative matrix to blood for the biological monitoring of inorganic lead. Toxicol Lett.

[44] Philpott CC (2014). Pumping iron. Elife.

[45] Gil F, Hernández AF, Márquez C, Femia P, Olmedo P, López-Guarnido O (2011). Biomonitorization of cadmium, chromium, manganese, nickel and lead in whole blood, urine, axillary hair and saliva in an occupationally exposed population. Sci Total Environ.

[46] Davis E, Bakulski KM, Goodrich JM, Peterson KE, Marazita ML, Foxman B (2020). Low levels of salivary metals, oral microbiome composition and dental decay. Sci Rep.

[47] Herman M, Golasik M, Piekoszewski W, Walas S, Napierala M, Wyganowska-Swiatkowska M (2016). Essential and toxic metals in oral fluid-a potential role in the diagnosis of periodontal diseases. Biol Trace Elem Res.

[48] Watanabe K, Tanaka T, Shigemi T, Hayashida Y, Maki K (2009). Mn and Cu concentrations in mixed saliva of elementary school children in relation to sex, age, and dental caries. J Trace Elem Med Biol.

[49] Quadras DD, Nayak USK, Kumari NS, Priyadarshini HR, Gowda S, Fernandes B (2019). In vivo study on the release of nickel, chromium, and zinc in saliva and serum from patients treated with fixed orthodontic appliances. Dent Res J (Isfahan).

[50] Basir L, Meshki R, Behbudi A, Rakhshan V (2019). Effects of restoring the primary dentition with stainless-steel crowns on children’s salivary nickel and chromium levels, and the associations with saliva pH: a preliminary before-after clinical trial. Biol Trace Elem Res.

[51] Planchart A, Green A, Hoyo C, Mattingly CJ (2018). Heavy metal exposure and metabolic syndrome: evidence from human and model system studies. Curr Environ Health Rep.

[52] Wilhelm M, Pesch A, Rostek U, Begerow J, Schmitz N, Idel H (2002). Concentrations of lead in blood, hair and saliva of German children living in three different areas of traffic density. Sci Total Environ.

[53] Apostolou A, Garcia-Esquinas E, Fadrowski JJ, McLain P, Weaver VM, Navas-Acien A (2012). Secondhand tobacco smoke: a source of lead exposure in US children and adolescents. Am J Public Health.

[54] Richter PA, Bishop EE, Wang J, Kaufmann R (2013). Trends in tobacco smoke exposure and blood lead levels among youths and adults in the United States: the National Health and Nutrition Examination Survey, 1999-2008. Prev Chronic Dis.

[55] Mannino DM, Albalak R, Grosse S, Repace J (2003). Second-hand smoke exposure and blood lead levels in U.S. children. Epidemiology.

[56] Salnikow K, Zhitkovich A (2008). Genetic and epigenetic mechanisms in metal carcinogenesis and cocarcinogenesis: nickel, arsenic, and chromium. Chem Res Toxicol.

[57] Borella P, Fantuzzi G, Aggazzotti G (1994). Trace elements in saliva and dental caries in young adults. Sci Total Environ.

[58] Duggal MS, Chawla HS, Curzon ME (1991). A study of the relationship between trace elements in saliva and dental caries in children. Arch Oral Biol.

[59] Hussein AS, Ghasheer HF, Ramli NM, Schroth RJ, Abu-Hassan MI (2013). Salivary trace elements in relation to dental caries in a group of multi-ethnic schoolchildren in Shah Alam, Malaysia. Eur J Paediatr Dent.

[60] Gray N, Halstead M, Gonzalez-Jimenez N, Valentin-Blasini L, Watson C, Pappas RS (2019). Analysis of toxic metals in liquid from electronic cigarettes. Int J Environ Res Public Health.

[61] Hess CA, Olmedo P, Navas-Acien A, Goessler W, Cohen JE, Rule AM (2017). E-cigarettes as a source of toxic and potentially carcinogenic metals. Environ Res.

[62] Hecht SS (2003). Tobacco carcinogens, their biomarkers and tobacco-induced cancer. Nat Rev Cancer.

[63] Sharma P, Sane N, Anand SD, Marimutthu P, Benegal V (2019). Assessment of cotinine in urine and saliva of smokers, passive smokers, and nonsmokers: Method validation using liquid chromatography and mass spectrometry. Indian J Psychiatry.

[64] Bessonneau V, Pawliszyn J, Rappaport SM (2017). The saliva exposome for monitoring of individuals’ health trajectories. Environ Health Perspect.

[65] Ladva CN, Golan R, Greenwald R, Yu T, Sarnat SE, Flanders WD (2017). Metabolomic profiles of plasma, exhaled breath condensate, and saliva are correlated with potential for air toxics detection. J Breath Res.

[66] Li Z, Sarnat JA, Liu KH, Hood RB, Chang CJ, Hu X (2022). Evaluation of the use of saliva metabolome as a surrogate of blood metabolome in assessing internal exposures to traffic-related air pollution. Environ Sci Technol.

