# Supplementary material for: Environmental tobacco smoke exposure is associated with increased levels of metals in children’s saliva
Source: J Expo Sci Environ Epidemiol. 2023 May 5;33(6):903–10. doi: 10.1038/s41370-023-00554-w (PMC10733142; doi:10.1038/s41370-023-00554-w)
Supplement: Supplementary file 1 — Supplementary Information [file 41370_2023_554_MOESM1_ESM.docx]

**Suppl Table 1. Correlations among salivary metals.**

Correlations among metals were determined using Spearman correlation analysis. The Bonferroni corrected alpha levels is 0.05/6=0.0083. Findings that were significant at the uncorrected alpha level (0.05) are marked with an asterisk. Substitution with the LLoD for those samples <LLoD has been done prior to analysis, with the exception of Pb and Ni, due to the high undetection rates for these metals (see Table 1 of main text).

**Suppl. Figure 1. The effects of saliva processing variables on metals measurements.**

The effects of a spiked-in known amount of each metal into saliva samples from n=5 normal control subjects was tested for variations due to saliva type (passive drool vs. swab), saliva processing method (centrifuged vs. uncentrifuged), storage conditions (-20C, 4C, room temperature for 24 hrs), or the number of freeze-thaw cycles (2-3). Differences in conditions for each metal were determined by Student’s t test (two-tailed; unpaired). **, P=0.005.
